# Contrast-enhanced CT-based radiomics model for differentiating risk subgroups of thymic epithelial tumors

**DOI:** 10.1186/s12880-022-00768-8

**Published:** 2022-03-06

**Authors:** Chunhai Yu, Ting Li, Xiaotang Yang, Ruiping Zhang, Lei Xin, Zhikai Zhao, Jingjing Cui

**Affiliations:** 1 Department of Radiology, Shanxi Province Cancer Hospital; Shanxi Hospital Affiliated to Cancer Hospital, Chinese Academy of Medical Sciences; Cancer Hospital Affiliated to Shanxi Medical University, Taiyuan, 030013 Shanxi People’s Republic of China; 2 Department of Nephrology, Taiyuan People’s Hospital, Taiyuan, 030001 Shanxi People’s Republic of China; 3 Huiying Medical Technology Co., Ltd.,, Room C103, B2, Dongsheng Science and Technology Park, HaiDian District, Beijing City, 100192 People’s Republic of China

**Keywords:** Radiomics, Computed tomography, Thymoma, Thymic carcinoma

## Abstract

**Background:**

To validate a contrast-enhanced CT (CECT)-based radiomics model (RM) for differentiating various risk subgroups of thymic epithelial tumors (TETs).

**Methods:**

A retrospective study was performed on 164 patients with TETs who underwent CECT scans before treatment. A total of 130 patients (approximately 79%, from 2012 to 2018) were designated as the training set, and 34 patients (approximately 21%, from 2019 to 2021) were designated as the testing set. The analysis of variance and least absolute shrinkage and selection operator algorithm methods were used to select the radiomics features. A logistic regression classifier was constructed to identify various subgroups of TETs. The predictive performance of RMs was evaluated based on receiver operating characteristic (ROC) curve analyses.

**Results:**

Two RMs included 16 and 13 radiomics features to identify three risk subgroups of traditional risk grouping [low-risk thymomas (LRT: Types A, AB and B1), high-risk thymomas (HRT: Types B2 and B3), thymic carcinoma (TC)] and improved risk grouping [LRT* (Types A and AB), HRT* (Types B1, B2 and B3), TC], respectively. For traditional risk grouping, the areas under the ROC curves (AUCs) of LRT, HRT, and TC were 0.795, 0.851, and 0.860, respectively, the accuracy was 0.65 in the training set, the AUCs were 0.621, 0.754, and 0.500, respectively, and the accuracy was 0.47 in the testing set. For improved risk grouping, the AUCs of LRT*, HRT*, and TC were 0.855, 0.862, and 0.869, respectively, and the accuracy was 0.72 in the training set; the AUCs were 0.778, 0.716, and 0.879, respectively, and the accuracy was 0.62 in the testing set.

**Conclusions:**

CECT-based RMs help to differentiate three risk subgroups of TETs, and RM established according to improved risk grouping performed better than traditional risk grouping.

**Supplementary Information:**

The online version contains supplementary material available at 10.1186/s12880-022-00768-8.

## Background

Thymic epithelial tumors (TETs) originate from the thymus and are the most common primary neoplasms in the anterior mediastinum, accounting for approximately 47% of cases [1]. Pathological subtypes of TETs were determined by the World Health Organization (WHO) in 2004, including thymomas (Types A, AB, B1, B2, and B3) and thymic carcinoma (TC), based on morphologic manifestations of epithelial cells and the ratio of lymphocytes to epithelial cells [2]. In 2014, the International Thymic Malignancy Interest Group (ITMIG) affirmed the description of WHO histologic subtypes of TETs [3]. The six different subtypes were divided into three risk subgroups according to increasing grade of malignancy: low-risk thymomas (LRT; Types A, AB and B1), high-risk thymomas (HRT; Types B2 and B3), and TC in 2004 [4]. It has been agreed that TC has a poorer prognosis and a higher recurrence rate than HRT and LRT. According to the different subgroups of TETs, different standardized and appropriate treatment options and methods of predicting the clinical course and prognosis of the disease are used for each patient by the clinical multidisciplinary team [5, 6]. Therefore, accurate and noninvasive identification of TETs before treatment, and even of the subgroups, is of clinical significance.

According to the National Comprehensive Cancer Network (NCCN) guidelines for thymomas and thymic carcinomas in 2021, chest contrast-enhanced CT (CECT) with contrast is still the first choice for imaging evaluation before treatment [7]. Chest CECT imaging can provide many general morphologic parameters. However, there are many overlapping features in the histological subgroups of TETs, and certain difficulties in distinguishing different subgroups may be encountered [8, 9]. Radiomics, a diagnostic technology based on radiomics signatures, has aroused increasing attention, mainly because it can extract different kinds and large quantities of high-throughput imaging features and transform medical images into mineable high-dimensional data [10, 11]. The subsequent quantitative analysis of these data can offer help in differential diagnosis, risk classification, predicting prognosis and efficacy evaluation of tumors based on different kinds of medical images [12–15]. Although several CT-based radiomics analyses have been used to identify the risk classification of thymic epithelial tumors, most studies were based on two-classification [16, 17]. Only one study was based on triple classification, and the accuracy of the clinical-semantic radiomics model (RM) in the risk assessment of three subgroups in the validation group was only 48.3% [18]. Therefore, radiomics research based on triple classification needs further research.

Previous studies have found that although type B1 thymomas are LRTs in terms of biological characteristics and invasive performance, their imaging features are more similar to those of types B2 and B3 thymomas [19]. In addition, the results of Kim et al. showed that the disease-free survival at 5 years of type B1, B2 and B3 thymomas was basically similar [20]. Therefore, we tried to regroup the six subtypes into three risk subgroups: LRT* (Types A and AB), HRT* (Types B1, B2, and B3), and TC. In this article, the subgroups were named traditional risk grouping (LRT, HRT, and TC) and improved risk grouping (LRT*, HRT*, and TC) to facilitate the description of articles and statistics of data.

This study aimed to build two CECT-based RMs and validate their predictive abilities in differentiating three different risk subgroups of TETs in the two simplified groups.

## Methods

### Patients

The retrospective study was approved by the institutional review board of Shanxi Province Tumor Hospital. The individual written informed consent was waived. The study included 179 patients with pathologically confirmed TETs in the anterior mediastinum from October 2012 to March 2021. Accurate pathological classifications were obtained in 164 patients, including 45 cases of biopsy and 119 cases of surgical resection, while not accurate pathological classifications were obtained in 15 patients, including 14 cases of biopsy and 1 case of surgical resection. All 164 patients who were included in this radiomics study underwent CECT scans before treatment. The inclusion criteria were as follows: (a) solid anterior mediastinal TETs; (b) lesions > 2.0 cm in diameter based on the longest diameter; (c) good-quality CECT images without movement artifacts; and (d) patients who did not undergo biopsy, treatment with chemotherapy, radiation therapy, or surgery before CT scan.

**Fig. 1:**
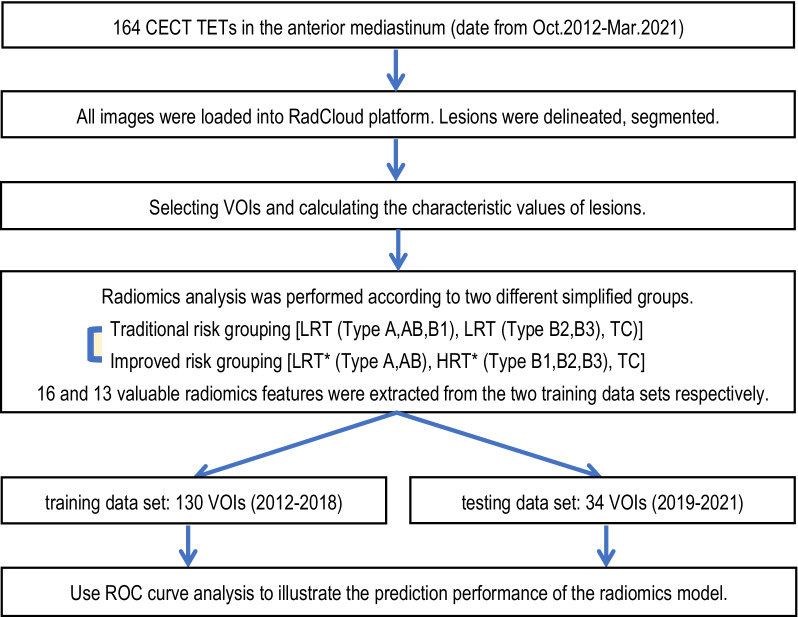
Radiomics analysis workflow. First, 164 TETs in the anterior mediastinum on CECT were collected. Second, image segmentation was used to delineate the TET lesions on the RadCloud platform, the volume of interest (VOIs) was checked manually, and the radiomics features of VOIs were calculated automatically. In addition, the two kinds of valuable radiomics features were extracted by the automated high-throughput feature analysis algorithm according to two different simplified groups in the training set. Finally, statistical analysis was applied, and ROC curve analysis was used to illustrate the prediction performance of RM for the risk subgroups of TETs

Determine the number of patients in the training set and test set according to the time. A total of 130 patients (approximately 79%, from 2012 to 2018) were designated as the training set, and 34 patients (approximately 21%, from 2019 to 2021) were designated as the testing set. The distribution of the training set and testing set of 164 patients is shown in Table 1. The workflow was shown in Fig. 1.

**Table 1:** The distribution of the training set and testing set of 164 patients

Data set (year)	Number (%)	WHO pathological subtypes						Traditional risk grouping			Improved risk grouping		
		Type A	Type AB	Type B1	Type B2	Type B3	TC	LRT	HRT	TC	LRT*	HRT*	TC
								(A/AB/B1)	(B2/B3)		(A/AB)	(B1/B2/B3)	
All data	164	15	19	26	34	24	46	60	58	46	34	84	46
(2012–2021)	(100%)	(9.1%)	(11.6%)	(15.9%)	(20.7%)	(14.6%)	(28.0%)	(36.6%)	(35.4%)	(28.0%)	(20.7%)	(51.2%)	(28.0%)
Traini ng set	130	13	14	21	26	20	36	48	46	36	27	67	36
(2012–2018)	(79%)	(10.0%)	(10.8%)	(16.2%)	(20.0%)	(15.4%)	(27.7%)	(36.9%)	(35.4%)	(27.7%)	(20.8%)	(51.5%)	(27.7%)
Testing set	34	2	5	5	8	4	10	12	12	10	7	17	10
(2019–2021)	(21%)	(6.9%)	(14.7%)	(14.7%)	(23.5%)	(11.8%)	(29.4%)	(35.3%)	(35.3%)	(29.4%)	(20.6%)	(50.0%)	(29.4%)

LRT, low-risk thymomas; HRT, high-risk thymomas; TC, thymic carcinoma

### CT images

The Digital Imaging and Communications in Medicine (DICOM) CECT images were scanned by a GE Discovery CT 750HD scanner (Waukesha, WI) and a GE lightspeed Healthcare CT scanner. Automatic tube current modulation techniques were adopted with the tube voltage set at 120 kVp. Before scanning, patients were instructed to hold their breath to avoid motion artifacts. The first series was a thorax noncontrast CT study (helical scan type, 100 kV and automatic mAs, the rotation time was 0.6 s, the slice thickness and interval were each 5 mm, the pitch was 1.375:1, the scanning field of view (SFOV) was 50 cm, and the matrix was 512*512); the scan range was from the thoracic inlet to the diaphragmatic level. A total of 50 to 120 mL (1 mL/kg weight) of contrast medium (iohexol, 300 mg/mL, iodine) was injected by using a pump injector at a rate of 3.0 mL/s. Venou phase scanning began 35 s after the trigger attenuation threshold (120 HU) achieved the level of the thoracic aorta. The scanning parameters were the same as those in the noncontrast CT study.

### Lesion delineation and segmentation

**Fig. 2:**
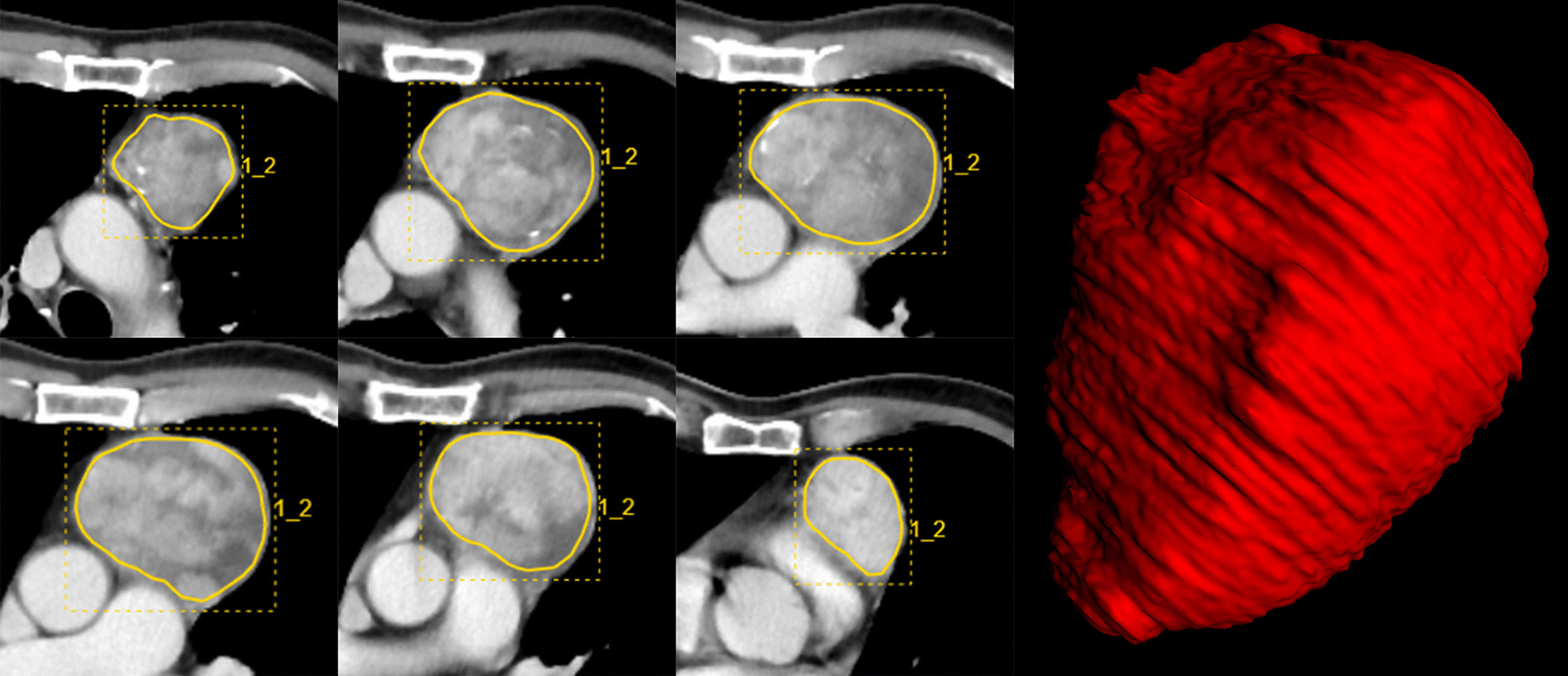
TET lesions segmentation. On all consecutive CECT images, the contour of the lesions was drawn manually along the edge of the lesions, and VOIs were automatically obtained

All DICOM CECT images were loaded into the RadCloud platform (Huiying Medical Technology Co., Ltd. https://mics.radcloud.cn). RadCould radiomics platform used open source code, which can be obtained online (https://readthedocs.org/projects/pyradiomics/downloads/). The region of interest (ROI) of the lesion was handcrafted layer by layer on 5 mm thick venous CECT images on the platform by a radiologist with 10 years of experience (X.L.). Volumes of interest (VOIs) were automatically calculated and generated (Fig. 2).

### Radiomics features

In total, 1409 quantitative imaging features were extracted from venous-phase CECT images with the RadCloud platform, which the feature extraction module is based on the “pyradiomics” (version 2.2.0, https://pyradiomics.readthedocs.io/) package in Python (Version 2.7). They were grouped into four categories. Category 1 covered the intensity features (including 18 descriptors) that quantitatively delineated the distribution of voxel intensities within the CT image through the basic metrics found in common. Category 2 (shape features) consists of 14 three-dimensional (3D) features that describe the geometric features of the target area, such as shape and size. Category 3 (texture features). The 75 features described the characteristics of voxel spatial distribution intensity levels and were divided into five types based on the gray level cooccurrence matrix (GLCM), gray size area band matrix (GLSZM), gray run length matrix (GLRLM), gray level dependence matrix (GLDM), and neighboring gray tone difference matrix (NGTDM). The above three categories all extracted features from the VOIs of the original image. Category 4 (higher-order features), with 1302 features, included the intensity and texture features that were derived from the wavelet transformation and the filters of the original image. In this study, a total of 14 filters were used for the filtering of the original image, including exponential, square, square root, logarithm, gradient, local binary pattern and wavelet (wavelet-LLL, wavelet-HHH, wavelet-HLL, wavelet-HHL, wavelet-LLH, wavelet-HLH, wavelet-LHL, wavelet-LHL, wavelet-LHH). Before feature extraction, the images were resampled to 1 * 1 * 1, and the gray-level normalization were applied for the standardization of the CT images.

### Radiomics feature selection and model establishment

All statistical analyses were performed in Python (Version 2.7) using “scitkit-learn” (V0.2 https://scikit-learn.org/stable/). Before feature selection, Z-Score was used for feature standardization. We used analysis of variance (ANOVA) and least absolute shrinkage and selection operator (LASSO) algorithm methods for feature selection to identify the optimal features. The cost function of LASSO method is:

$$\mathop {\min }\limits_{w} \frac{1}{2n}\left\| {Xw - y} \right\|_{2}^{2} + \alpha \left\| w \right\|_{1}$$

where *X* is the matrix of radiomic features, y is the vector of the sample labels, n is the number of samples, w is the coefficient vector of the regression model, *and* $\alpha \left\| w \right\|_{1}$ is the LASSO penalty with the constant $\alpha$ and the $l_{1}$-norm of coefficient vector $\left\| w \right\|_{1}$.

We used a logistic regression (LR) classifier on CECT selected features. A logistic function or logistic curve is a common "S" shape (sigmoid curve), with the following equation:

$$y = \frac{L}{{1 + \exp \left( { - k\left( {x - x_{0} } \right)} \right)}}$$

where e is the natural logarithm base (also known as Euler's number),$x_{0}$ is the *x*-value of the sigmoid's midpoint, *L* is the curve's maximum value, and *k* is the steepness of the curve.

The cost function of LR as following:

$$\mathop {\min }\limits_{w,c} \frac{1}{2}w^{T} w + \mathop \sum \limits_{i = 1}^{n} \log \left( {\exp \left( { - y_{i} \left( {X_{i}^{T} + c} \right)} \right) + 1} \right)$$

where the parameters are the same as the cost function for LASSO [21].

### Assessment of inter- and intraclass correlation coefficients (ICCs)

To ensure reproducibility of radiomics feature extraction, we employed inter- and intraclass correlation coefficients (ICCs) for assessing the intra- and interobserver agreement of VOI delineation. Thirty lesions were selected randomly by statistical software. After 1 month, another radiologist (Z.Z.K) with 13 years of clinical experience used the same method to extract radiomics features. An ICC > 0.75 was considered to represent good agreement.

### Predictive performance of RMs after machine learning

Receiver operating characteristic (ROC) curve analysis was used to evaluate the prediction ability of the two different RMs. The optimal cutoff value was selected as the point when both the sensitivity and specificity were maximal. The area under the curve (AUC) and accuracy were calculated in both the training and testing sets. The three indicators were P (precision = true positives/(true positives + false positives)), R (recall = true positives/(true positives + false negatives)), and f1-score (f1-score = P × R × 2/(P + R)), to evaluate the performance of the LR classifier. The clinical benefits of two RMs were estimated by decision curve analyses, and the goodness-of-fits of the two RMs were evaluated by calibration curves. They were accomplished with R 4.0.3 (www.R-project.org/).

## Results

### General data

A total of 164 patients (mean age: 54 ± 10.33 years, age range: 24–78 years) with TETs for CECT scans were enrolled: 78 men and 86 women. According to the histological and immunohistochemical results, with regard to WHO pathological subtypes, there were 15 (9.1%) Type A patients, 19 (11.6%) Type AB, 26 (15.9%) Type B1, 34 (20.7%) Type B2, 24 (14.6%) Type B3, and 46 (28.0%) TC (including 4 cases of thymic carcinoid) (Table 1).

### Radiomics features selection

**Fig. 3:**
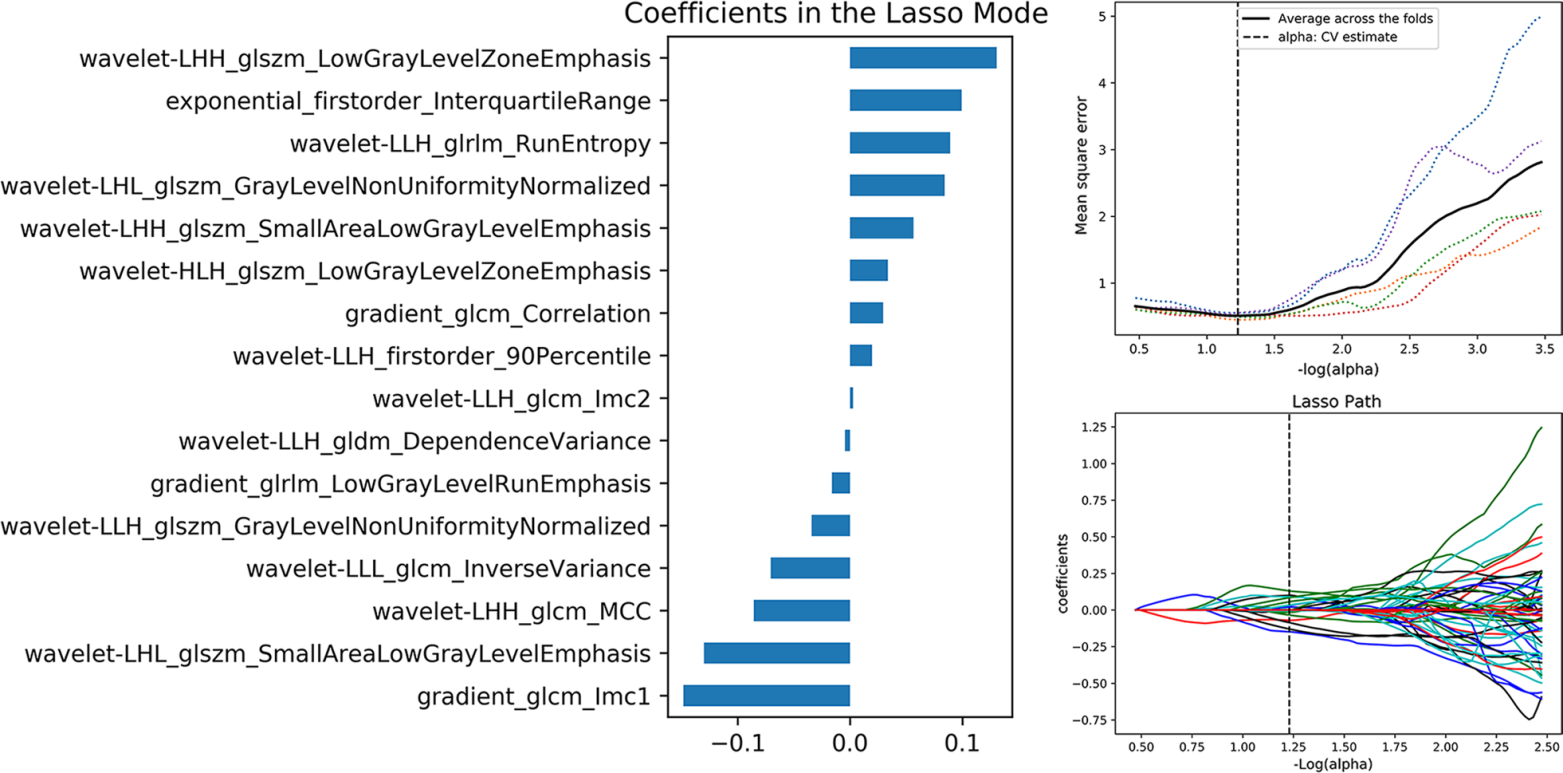
Valuable radiomics feature selection of traditional risk grouping [LRT (Types A, AB and B1), HRT (Types B2 and B3), TC)] using LASSO regression. The optimal value of the lasso tuning parameter (alpha = 1.241) was found, and 16 features that corresponded to the optimal alpha value were extracted following coefficients on CECT images

**Fig. 4:**
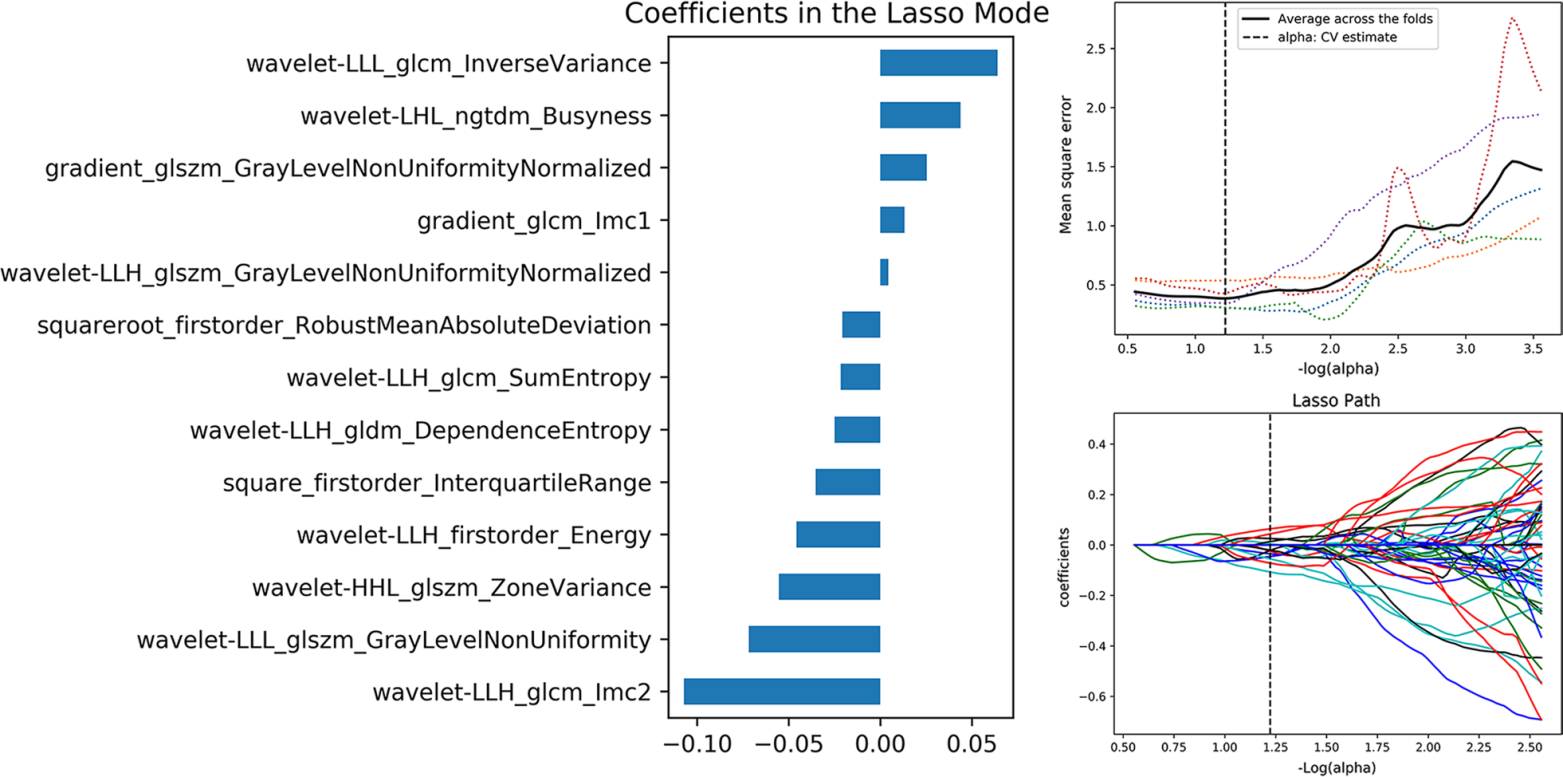
Valuable radiomics feature selection of improved risk grouping [LRT* (Types A and AB), HRT* (Types B1, B2 and B3), TC] using LASSO regression. The optimal value of the lasso tuning parameter (alpha = 1.239) was found, and 13 features that corresponded to the optimal alpha value were extracted following coefficients on CECT images

The inter- and intraobserver reproducibility of feature extraction was achieved with ICCs > 0.75 between the two different radiologists. The 16 and 13 features were selected by the ANOVA and Lasso algorithm method, and the corresponding optimal values of the lasso tuning parameter (alpha) were 1.241 and 1.239, respectively. Then, the two RMs included 16 and 13 radiomics features to identify three different subgroups of TETs according to traditional risk grouping [LRT (Types A, AB and B1), LRT (Types B2 and B3), TC] and improved risk grouping [LRT* (Types A and AB), HRT* (Types B1, B2 and B3), TC], respectively (Figs. 3, 4).

The features in the two RMs were all high-order features without any intensity, shape or texture features, four of which were the same: wavelet-LLL_glcm_InverseVariance, wavelet-LLH_glcm_Imc2, gradient_glcm_Imc1 and wavelet-LLH_glszm_GrayLevelNonUniformityNormalized.

### Diagnostic performance of the two RMs

**Fig. 5:**
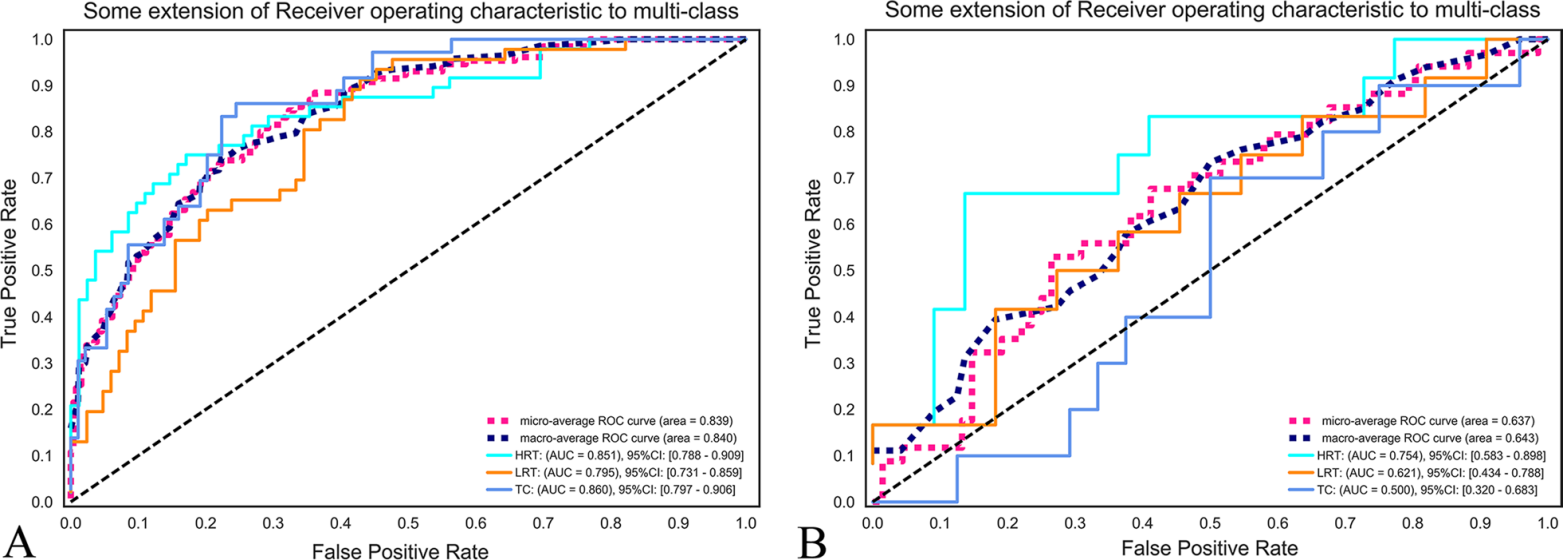
Receiver operating characteristic curve (ROC) on CECT-based RM according to traditional risk grouping [LRT (Types A, AB and B1), HRT (Types B2 and B3), TC)]. **a** The 16-feature RM was trained in the training set with the LR classifier. The areas under the ROC curve (AUCs) of LRT, HRT, and TC were 0.795, 0.851, and 0.860, respectively. **b** The 16-feature RM was tested in the training set with the LR classifier. The AUCs of LRT, HRT, and TC were 0.621, 0.754, and 0.500, respectively

**Fig. 6:**
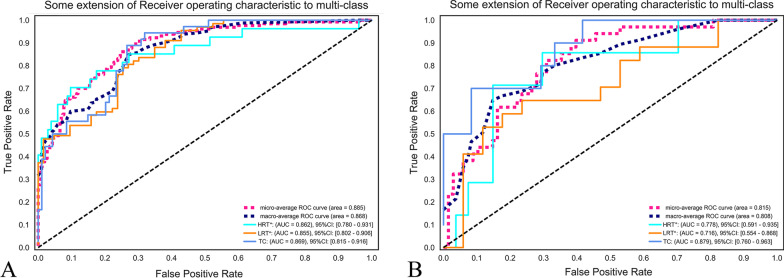
Receiver operating characteristic curve (ROC) on CECT-based RM according to improved risk grouping [LRT* (Types A and AB), HRT* (Types B1, B2 and B3), TC]. **a** The 13-feature RM was trained in the training set with the LR classifier. The areas under the ROC curve (AUCs) of LRT, HRT, and TC were 0.855, 0.862, and 0.869, respectively. **b** The 13-feature RM was tested in the training set with the LR classifier. The AUCs of LRT, HRT, and TC were 0.778, 0.716, and 0.879, respectively

**Fig. 7:**
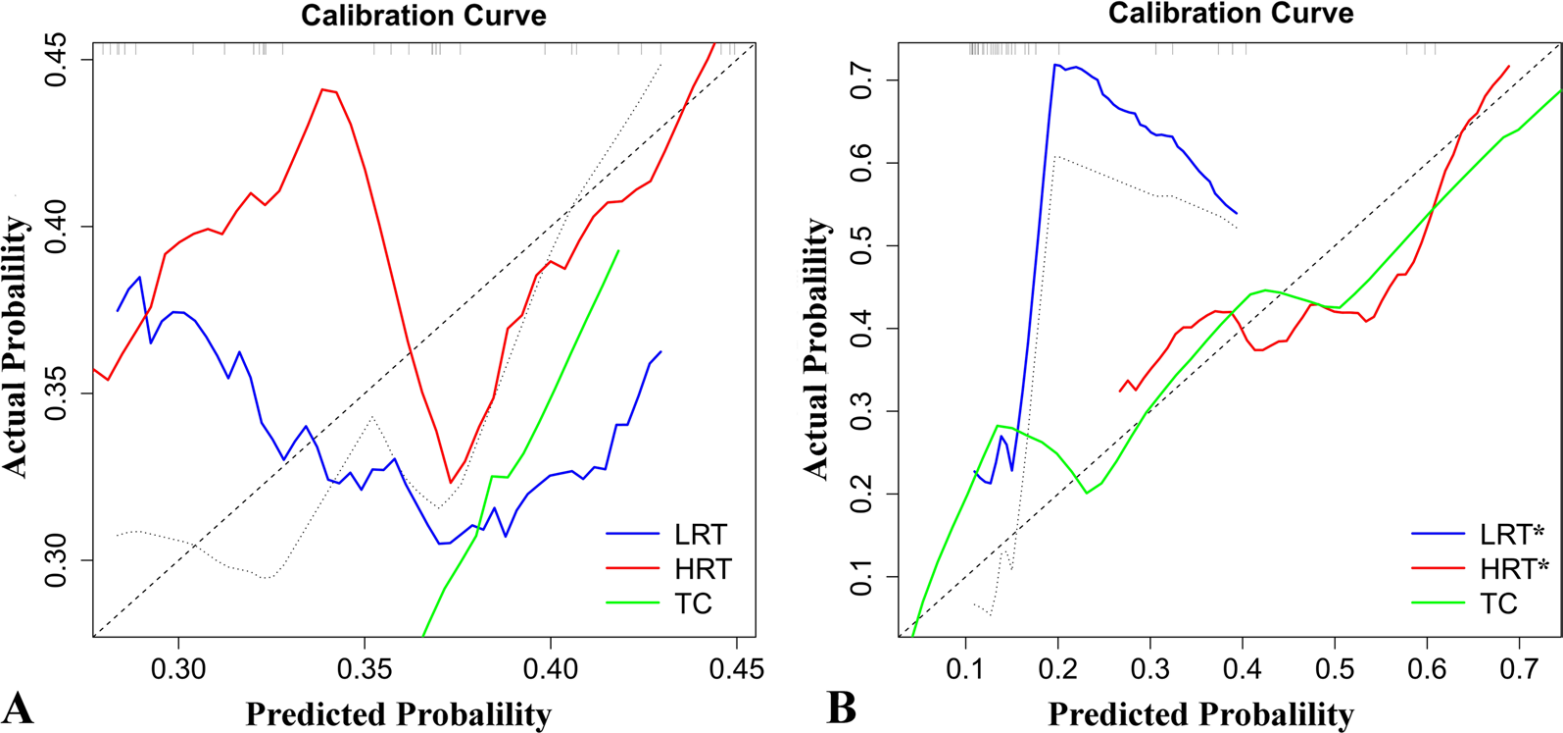
The calibration curves of the two RMs in the testing sets respectively. **a** For the traditional risk grouping [LRT (Types A, AB and B1), HRT (Types B2 and B3), TC)], the prediction performance of RM for LRT, HRT and TC did not show satisfactory consistency with the actual risk level. **b** For the traditional risk grouping [LRT* (Types A and AB), HRT* (Types B1, B2 and B3), TC], the prediction performance of RM for HRT* and TC showed satisfactory consistency with the actual risk level

**Fig. 8:**
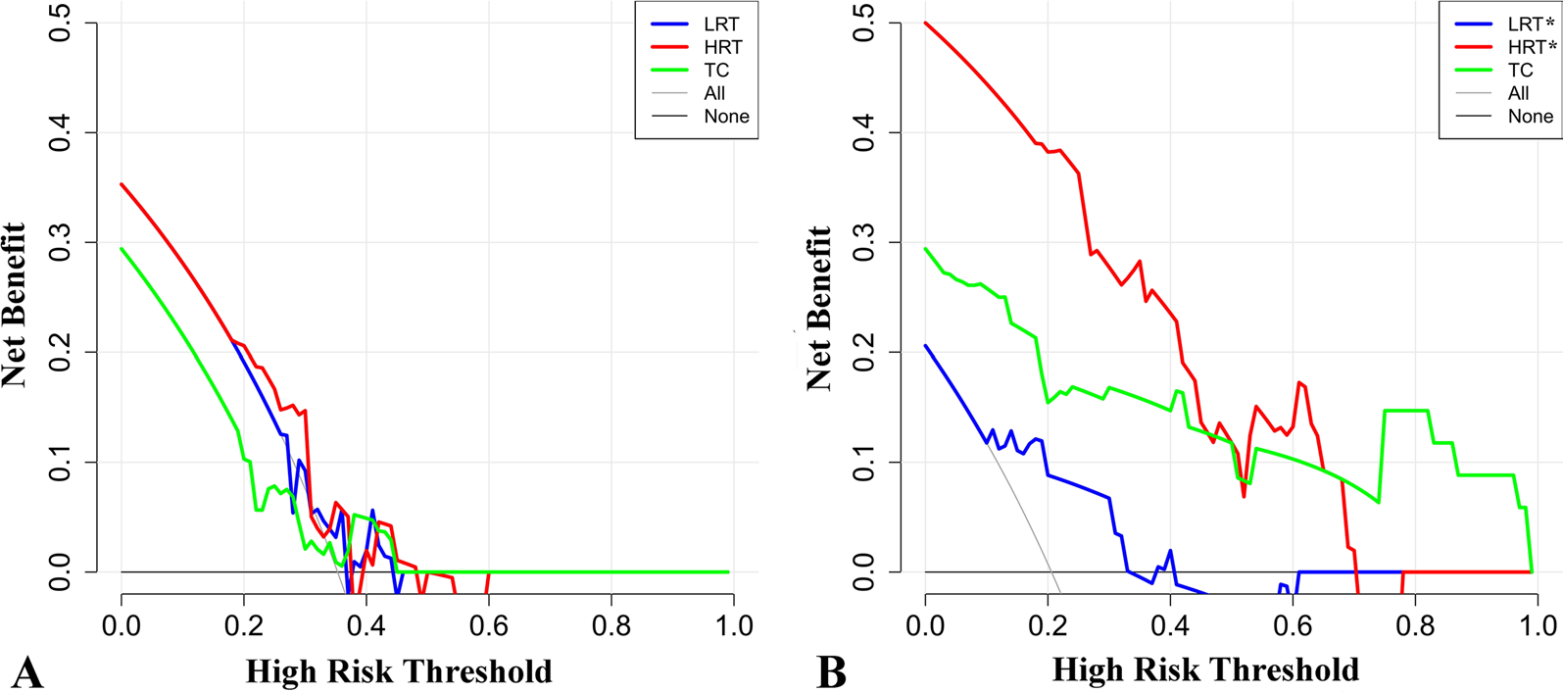
The decision curve analyses of the two RMs in the testing sets respectively. **a** The RM according to the traditional risk grouping [LRT (Types A, AB and B1), HRT (Types B2 and B3), TC)] had general clinical utility for LRT, HRT and TC. **b** The RM according to the improved risk grouping [LRT* (Types A and AB), HRT* (Types B1, B2 and B3), TC] had good clinical utility for HRT* and TC

The 16- and 13-feature RMs were trained with the LR classifier on CECT images, and the ROC curve analysis results are shown in Figs. 5 and 6. In the training set of traditional risk grouping, the areas under the ROC curve (AUCs) of LRT, HRT, and TC were 0.795, 0.851, and 0.860, respectively, and the accuracy was 0.65; in the testing set, the AUCs were 0.621, 0.754, and 0.500, respectively, and the accuracy was 47%. In the training set of improved risk grouping, the AUCs of LRT*, HRT*, and TC were 0.855, 0.862, and 0.869, respectively, the accuracy was 0.72, and in the testing set, the AUCs were 0.778, 0.716, and 0.879, respectively, and the accuracy was 0.62. For the testing set, the AUC of TC in improved risk grouping was 0.879, which was significantly larger than 0.500 in traditional risk grouping (Table 2). Additional file 1: Tables S1–S4 showed the confusion matrices. The calibration curves showed that the predicted performance of RM according to the improved risk grouping for HRT* and TC were in satisfactory agreement with the actual risk level, while the performance of the RM according to the traditional risk grouping was unsatisfactory (Fig. 7). In addition, Analyses of decision curves showed that the RM according to the improved risk grouping for HRT* and TC obtained higher clinical utility (Fig. 8).

**Table 2:** The prediction performance of the two RMs

Simplified groups		Subgroups	AUC (95%CI)	Accuracy	Precision	Recall	F1-score
Traditional risk grouping	Training set	LRT (A,AB,B1)	0.795 (0.731–0.859)	0.65	0.73	0.67	0.70
		HRT (B2,B3)	0.851 (0.788–0.900)	0.65	0.63	0.57	0.60
		TC	0.860 (0.797–0.906)	0.65	0.58	0.72	0.64
	Testing set	LRT (A,AB,B1)	0.621 (0.434–0.768)	0.47	0.70	0.58	0.64
		HRT (B2,B3)	0.754 (0.583–0.898)	0.47	0.46	0.50	0.48
		TC	0.500 (0.320–0.683)	0.47	0.27	0.30	0.29
Improved risk grouping	Training set	LRT* (A,AB)	0.855 (0.802–0.906)	0.72	0.73	0.59	0.65
		HRT* (B1,B2,B3)	0.862 (0.780–0.931)	0.72	0.74	0.85	0.79
		TC	0.869 (0.815–0.916)	0.72	0.65	0.56	0.60
	Testing set	LRT* (A,AB)	0.716 (0.554–0.868)	0.62	0.40	0.29	0.33
		HRT* (B1,B2,B3)	0.778 (0.591–0.935)	0.62	0.61	0.82	0.70
		TC	0.879 (0.760–0.983)	0.62	0.83	0.50	0.62

RM, radiomics model; LRT, low-risk thymomas; HRT, high-risk thymomas; TC, thymic carcinoma; AUC, area under the curve

## Discussion

This study built two RMs based on CECT images using LASSO to extract the features and LR as the classifier to identify three different subgroups of TETs. After machine learning, the 13-feature RM (accuracy = 0.62) established according to improved risk grouping [LRT* (Types A and AB), HRT* (Types B1, B2 and B3), TC] showed a better predictive performance than the 16-feature RM (accuracy = 0.47) established according to traditional risk grouping [LRT (Types A, AB and B1), HRT (Types B2 and B3), TC] in the test set.

Recently, six popular machine learning algorithms have been used to construct RMs: k-nearest neighbor (KNN), support vector machine (SVM), eXtreme Gradient Boosting (XGBoost), random forest (RF), logistic regression (LR), and decision tree (DT). Among them, the results using the LR algorithm were the most ideal in many CT-based radiomics studies to predict different risk subgroups of TETs or thymomas [18, 21, 22]. Therefore, in this study, we only chose LR algorithm. In this study, the prediction accuracy of 16-feature RM according to traditional risk grouping was not ideal (only 0.47), which was basically consistent with the research results (0.45) of Liu et al. [18] in the testing set. Therefore, it can be seen from our and Liu et al.'s studies that the ability of CT-based RM to distinguish the three conventional risk groups of TETs was not ideal.

Several studies have shown that although type B1 thymoma belongs to LRT, its conventional CECT findings overlap with type B2 and B3 thymomas in HRT to a certain extent, especially with type B2 thymoma [9, 19]. At the same time, a study showed that the prognosis of type B1 thymoma is not significantly different from that of type B2 and B3 thymomas [20]. Therefore, based on the above contradictions, we propose the idea of regrouping, and we hypothesized that regrouping may be more conducive to the identification of TETs. To the best of our knowledge, this is the first study to propose the concept of improved risk grouping of TETs. In this study, we found that the prediction accuracy of 13-feature RM according to improved risk grouping was 0.62, which was higher than the 0.45 of the simple CECT-based model and the 0.48 of the CECT-based clinical-semantic-radiomics model of Liu et al. [18] in the testing set. The results of this study verified our hypothesis. In pathology, type B thymomas apparently represent a continuum from B1 to B3 thymomas, which shows a spectrum of lymphocyte to epithelial predominance [23]. It can also be understood that the pathological similarity between type B1 thymoma and type B2 thymoma is higher than that between type B1 thymoma and type A or AB thymoma. Therefore, pathologists may overlap in the diagnosis of type B1 and B2 thymomas (approximately 15% disagreement) [3]. This pathological manifestation may explain the phenomenon that there was a certain overlap between type B1 thymoma and type B2 and B3 thymomas on conventional CT features, and it is also a feasible basis for regrouping. Therefore, we applied the improved risk grouping method to fundamentally reduce the interference of type B1 thymoma in LRT and HRT, and the established RM improved the accuracy of diagnosis. In this study, for the improved risk grouping, the performance of the CECT-based RM also declined when moving from training set to testing set (from 0.72 to 0.62). Significant TET atypia should be one of the main reasons for the general decline of performance. We also found that the AUC of TC according to improved risk grouping was 0.879, which was significantly larger than 0.500 according to traditional risk grouping in the testing set. This indicated that the RM established according to the improved risk grouping method may have a higher accuracy in predicting the risk of TC. We speculated that the reason may be that the extracted valuable radiomics features were more specific for TC or that some thymomas in LRT* and HRT* were very similar in pathological manifestations.

The 3D analysis of the whole lesion could reflect the heterogeneity of the tumor more representative and provide more comprehensive information. Chaddad et al. [24] found that a 3D wavelet transform can distinguish colorectal cancer classification, which has higher accuracy and sensitivity than 2D wavelet transform. Therefore, we manually depicted ROIs along the lesion contour on each image and converted ROIs to VOIs. Finally, there were 11 and 9 3D-wavelet texture features in the two RMs, respectively. In our study, there was no shape feature in any of the extracted features in the two RMs, indicating that the shape features were not significantly different in the three different risk subgroups of TETs. The results of Han et al.'s conventional CT imaging to identify different risks of TETs showed that tumor size and contour significantly differed between LRT and HRT [25]. Our results were inconsistent with these results, which might be due to the relatively small number of cases, especially type A and AB thymomas.

Chest CECT was the first choice of imaging evaluation before treatment for TETs. In this study, the images with 5 mm thickness in the venous phase of conventional CECT were used for radiomics analysis because the image stability in the venous phase was better than that in the arterial phase. In the arterial phase, the concentration of contrast medium in the superior vena cava or brachiocephalic vein was quite high, and the adjacent area had obvious artifacts, which may affect the display of lesions. Wang et al. [26] used radiomics based on CECT images and noncontrast-enhanced CT (NECT) images to identify high-risk and low-risk thymomas with similar AUCs. We did not use the NECT image because in some of the NECT images, the obvious artifact in the lesion may affect the authenticity of the tumor heterogeneity, and the unclear edge is not conducive to the segmentation of the lesion. Therefore, we think that radiomics analysis based on CECT and 3D segmentation of all lesions may have broader application prospects for the evaluation of TETs. According to the improved risk grouping method, we only selected the images with a 5 mm thickness of the venous phase as the training set, segmented them to generate VOIs, and used LR as the classifier to extract features and establish the most simplified RM. After machine learning, the prediction accuracy of the test set was significantly higher than that of the CECT-based clinical-semantic-radiomics model of Liu et al. [18]. This indicated that improved risk grouping may have potential clinical popularization and application value. In addition, several studies have shown that the iodine concentration (IC) value of dual-energy CT (DECT) is valuable for distinguishing different risks of TETs [27, 28]. The radiomics evaluation of TETs based on DECT images combined with IC values is worthy of further study.

We know that only when patients obtain accurate pathological diagnosis results can a multidisciplinary diagnosis and treatment team give them the most appropriate treatment plan [29]. Although pathological diagnosis is the gold standard, not all patients can obtain a specific pathological diagnosis after biopsy. Similarly, we found that 15 patients with TETs did not obtain accurate pathological classification during our follow-up. For patients who could not obtain accurate pathological diagnosis results in time, we could use RM to evaluate their risk level before treatment and provide a multidisciplinary diagnosis and treatment team with suggestions on the tumor risk level. Our RM may also have important value for the risk assessment of TET patients without specific pathological classification.

This study had some limitations. First, individual medical centers were included in the study, and the number of cases was small. Combining multiple centers with a larger number of patients will be needed to verify our results. Second, although our study was a retrospective cohort study, there was selection bias. Third, to compare the prediction performance of the two RMs, cross validation was not used in this study. We grouped the data according to time, which may avoid the selection bias caused by machine learning to a certain extent. Further research is needed to verify our results. In addition, it was time-consuming and subjective to draw the contour manually. Therefore, it is necessary to develop a more efficient and accurate method of image contour drawing.

## Conclusions

Our study established a simple RM established based only on venous CECT images to distinguish the three risk subgroups [low-risk thymoma (Types A, AB and B1), high-risk thymoma (Types B2 and B3), thymic carcinoma] of TETs. If type B1 thymoma is reclassified as high-risk thymoma, RM established according to the improved grouping mode may have higher accuracy in predicting the three risk subgroups.

## Supplementary Information

**Additional file 1.** **Supplementary Table 1.** The confusion matrix of the training set for the traditional risk grouping. **Supplementary Table 2.** The confusion matrix of the testing set for the traditional risk grouping. **Supplementary Table 3.** The confusion matrix of the training set for the improved risk grouping. **Supplementary Table 4.** The confusion matrix of the testing set for the improved risk grouping.

## Data Availability

The datasets generated and/or analysed during the current study are not publicly available due to patient privacy protection, but are available from the corresponding author on reasonable request.

## Abbreviations

TETs: Thymic epithelial tumors
TC: Thymic carcinoma
LRT: Low-risk thymoma
HRT: High-risk thymoma
CECT: Contrast-enhanced CT
RM: Radiomics model
VOI: Volume of interest
GLCM: Gray level co-occurrence matrix
GLSZM: Gray size area band matrix
GLRLM: Gray run length matrix
GLDM: Gray level dependence matrix
NGTDM: Neighbouring gray tone difference matrix
LASSO: Least absolute shrinkage and selection operator
LR: Logistic regression
