# Supplementary material for: Contrast-enhanced CT-based radiomics model for differentiating risk subgroups of thymic epithelial tumors
Source: BMC Med Imaging. 2022 Mar 6;22:37. doi: 10.1186/s12880-022-00768-8 (PMC8898532; doi:10.1186/s12880-022-00768-8)
Supplement: Supplementary file 1 — Additional file 1. Supplementary Table 1. The confusion matrix of the training set for the traditional risk grouping. Supplementary Table 2. The confusion matrix of the testing set for the traditional risk grouping. Supplementary Table 3. The confusion matrix of the training set for the improved risk grouping. Supplementary Table 4. The confusion matrix of the testing set for the improved risk grouping. [file 12880_2022_768_MOESM1_ESM.docx]

**Supplementary Tables**

Supplementary Table 1 The confusion matrix of the training set for the traditional risk grouping.

| confusion matrices of the training set (n=130) | | CECT-based RM prediction | | | Recall |
| --- | --- | --- | --- | --- | --- |
|  |  | LRT | HRT | TC |  |
| Traditional Risk Grouping | LRT (A,AB,B1) | 32 | 9 | 7 | 0.67 |
|  | HRT (B2,B3) | 8 | 26 | 12 | 0.57 |
|  | TC | 4 | 6 | 26 | 0.72 |
| Precision | | 0.73 | 0.63 | 0.58 |  |

LRT, low-risk thymomas; HRT, high-risk thymomas; TC, thymic carcinoma;

CECT, contrast-enhanced CT; RM, radiomics model.

Supplementary Table 2 The confusion matrix of the testing set for the traditional risk grouping.

| confusion matrices of the testing set (n=34) | | CECT-based RM prediction | | | Recall |
| --- | --- | --- | --- | --- | --- |
|  |  | LRT | HRT | TC |  |
| Traditional Risk Grouping | LRT (A,AB,B1) | 7 | 2 | 3 | 0.58 |
|  | HRT (B2,B3) | 1 | 6 | 5 | 0.50 |
|  | TC | 2 | 5 | 3 | 0.30 |
| Precision | | 0.70 | 0.46 | 0.27 |  |

LRT, low-risk thymomas; HRT, high-risk thymomas; TC, thymic carcinoma;

CECT, contrast-enhanced CT; RM, radiomics model.

Supplementary Table 3 The confusion matrix of the training set for the improved risk grouping.

| confusion matrices of the training set (n=130) | | CECT-based RM prediction | | | Recall |
| --- | --- | --- | --- | --- | --- |
|  |  | LRT | HRT | TC |  |
| Improved Risk Grouping | LRT* (A,AB) | 16 | 9 | 2 | 0.59 |
|  | HRT* (B1,B2,B3) | 1 | 57 | 9 | 0.85 |
|  | TC | 5 | 11 | 20 | 0.56 |
| Precision | | 0.73 | 0.74 | 0.65 |  |

LRT, low-risk thymomas; HRT, high-risk thymomas; TC, thymic carcinoma;

CECT, contrast-enhanced CT; RM, radiomics model.

Supplementary Table 4 The confusion matrix of the testing set for the improved risk grouping.

| confusion matrices of the testing set (n=34) | | CECT-based RM prediction | | | Recall |
| --- | --- | --- | --- | --- | --- |
|  |  | LRT | HRT | TC |  |
| Traditional Risk Grouping | LRT* (A,AB) | 2 | 4 | 1 | 0.29 |
|  | HRT* (B1,B2,B3) | 3 | 14 | 0 | 0.82 |
|  | TC | 0 | 5 | 5 | 0.50 |
| Precision | | 0.40 | 0.61 | 0.83 |  |

LRT, low-risk thymomas; HRT, high-risk thymomas; TC, thymic carcinoma;

CECT, contrast-enhanced CT; RM, radiomics model.
